# Quality Control of Photosystem II: The Mechanisms for Avoidance and Tolerance of Light and Heat Stresses are Closely Linked to Membrane Fluidity of the Thylakoids

**DOI:** 10.3389/fpls.2016.01136

**Published:** 2016-08-02

**Authors:** Yasusi Yamamoto

**Affiliations:** Graduate School of Natural Science and Technology, Okayama UniversityOkayama, Japan

**Keywords:** photosystem II, thylakoid, light stress, heat stress, membrane fluidity, D1 protein, protein aggregation, lipid peroxidation

## Abstract

When oxygenic photosynthetic organisms are exposed to excessive light and/or heat, Photosystem II is damaged and electron transport is blocked. In these events, reactive oxygen species, endogenous radicals and lipid peroxidation products generated by photochemical reaction and/or heat cause the damage. Regarding light stress, plants first dissipate excessive light energy captured by light-harvesting chlorophyll protein complexes as heat to avoid the hazards, but once light stress is unavoidable, they tolerate the stress by concentrating damage in a particular protein in photosystem II, i.e., the reaction-center binding D1 protein of Photosystem II. The damaged D1 is removed by specific proteases and replaced with a new copy produced through *de novo* synthesis (reversible photoinhibition). When light intensity becomes extremely high, irreversible aggregation of D1 occurs and thereby D1 turnover is prevented. Once the aggregated products accumulate in Photosystem II complexes, removal of them by proteases is difficult, and irreversible inhibition of Photosystem II takes place (irreversible photoinhibition). Important is that various aspects of both the reversible and irreversible photoinhibition are highly dependent on the membrane fluidity of the thylakoids. Heat stress-induced inactivation of photosystem II is an irreversible process, which may be also affected by the fluidity of the thylakoid membranes. Here I describe why the membrane fluidity is a key to regulate the avoidance and tolerance of Photosystem II on environmental stresses.

## Introduction – How Do the Chloroplasts Respond to Excessive Light and Heat?

**FIGURE 1:**
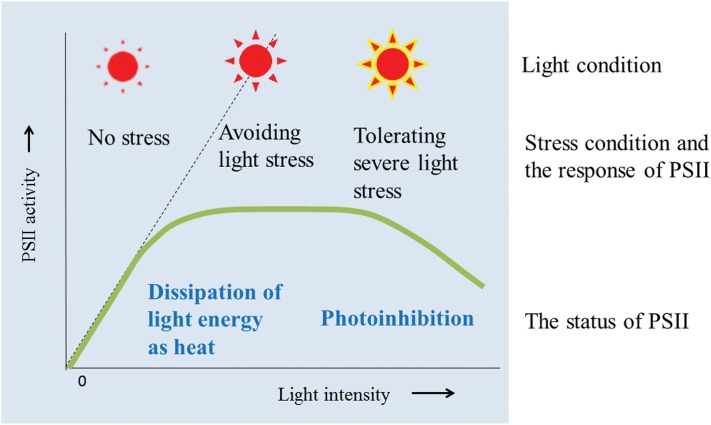
**Light response curve for PSII activity and the events that occur in the thylakoids when the thylakoids avoid and tolerate light stress.** Under low light, PSII activity increases with increasing light intensity. Under high light conditions, chloroplasts first try to avoid the light stress by dissipating the excessive light energy as heat (qE of NPQ). However, when the light intensity increases, photoinhibition of PSII becomes apparent. Irreversible aggregation of PSII proteins occurs under severe light stress conditions, which induces irreversible photoinhibition of PSII.

Plants are exposed to various stresses under natural environmental conditions. Adequate light and temperature are necessary for healthy growth of plants, but when either the light or temperature is extreme, it becomes a stress factor to plants. Concerning sunlight, both light quality and quantity change depending on the time and weather of day. To capture light energy efficiently under ever-changing environmental conditions, the chloroplasts often move in the cell, whereas in the chloroplasts, light-harvesting chlorophyll-protein complexes migrate on the thylakoid membranes to receive light energy. Conversely, when the chloroplasts or more directly the chlorophyll-protein complexes receive excessive light from the sun, they avoid or tolerate the light stress using both short and long term strategies (**Figure 1**). Under natural conditions, high or low temperature stress is superimposed on the light stress, which makes the situation more complicated and difficult. Here, I focus on the mechanisms for avoidance and tolerance of light and heat stresses at the thylakoids in higher plant chloroplasts, with special emphasis on importance of membrane fluidity of the thylakoids, which enables the avoidance and tolerance mechanisms to work efficiently.

## Avoidance of Light Stress – A Mechanism to Dissipate Extra Light Energy As Heat

When chloroplasts are irradiated by excessive light, Photosystem (PS) II core and the light-harvesting complexes of PSII (LHCII), which are associated with each other in the thylakoid membranes under normal light conditions, are disconnected and energy transfer from LHCII to PSII core is prevented (Yamamoto et al., 2008). Moreover, relocation and/or reorientation of LHCII and PSII complexes take place in the thylakoid membranes in order to decrease energy transfer from LHCII to PSII core (Goral et al., 2010; Herbstova et al., 2012; Yamamoto et al., 2014). These molecular rearrangements upon strong illumination can make the excessive excitation energy captured in LHCII dissipate as heat, and in this respect, reversible aggregation of LHCII plays an important role (Horton et al., 1991; Niyogi, 1999). This phenomenon has been monitored using pulse amplitude modulation (PAM) fluorometers (Walz, Germany; Schreiber et al., 1986) and is referred to as energy-dependent quenching (qE) in non-photochemical quenching of chlorophyll fluorescence (NPQ; Horton et al., 1996; Niyogi, 1999). In this process, the so-called xanthophyll cycle is activated through de-epoxidation of a xanthophyll pigment violaxanthin to zeaxanthin catalyzed by the enzyme violaxanthin-deepoxidase (VDE; Yamamoto et al., 1972; Demmig-Adams, 1990). A PsbS protein in land plants (Li et al., 2000) and a LHCSR in green algae (Peers et al., 2009), which are both the members of LHC gene family, sense the luminal pH and may induce specific aggregation of LHCII through protonation of the sensor proteins. These processes require membrane fluidity of the thylakoid membranes, which ensures movement of the necessary components on the membranes. For example, violaxanthin is released from LHCII and moves in the thylakoid membrane to VDE for de-epoxidation. It is converted to zeaxanthin by VDE and goes back to the LHCII. This xanthophyll conversion process is diffusion limited (Jahns et al., 2009; Kirchhoff, 2014). Structural flexibility of LHCII, assembly of the supercomplexes on the thylakoids, and structural change of the whole thylakoids studied by CD spectra etc. are reviewed recently (Garab, 2014), and these processed are also dependent on the membrane fluidity. Besides the total membrane fluidity of the thylakoids, local fluidity of the membranes may change during excessive illumination, in particular in the grana where PSII-LHCII supercomplex is abundant and active movement of PSII and LHCII is necessary (Yamamoto et al., 2013; Kirchhoff, 2014; Yamamoto et al., 2014).

## Tolerance of Light Stress – A Mechanism to Concentrate Photodamage on the D1 Protein and Enhance Protein Turnover

### Reversible Photoinhibition

PhotosystemII is brought into a severe stress condition when light intensity increases furthermore and avoidance of the light stress is difficult (Kyle et al., 1984; Powles, 1984). In this state, a tolerance mechanism works, which concentrates photodamage to the reaction center-binding D1 protein (D1) and stimulates the turnover of the protein (Mattoo et al., 1981; Ohad et al., 1984; Barber and Andersson, 1992; Aro et al., 1993). The injured PSII-LHCII supercomplex is disorganized and the damaged D1 is replaced by a new copy. Thus, the photoinhibition under these conditions is reversible and is referred to as ‘*reversible photoinhibition*’ herein.

**FIGURE 2:**
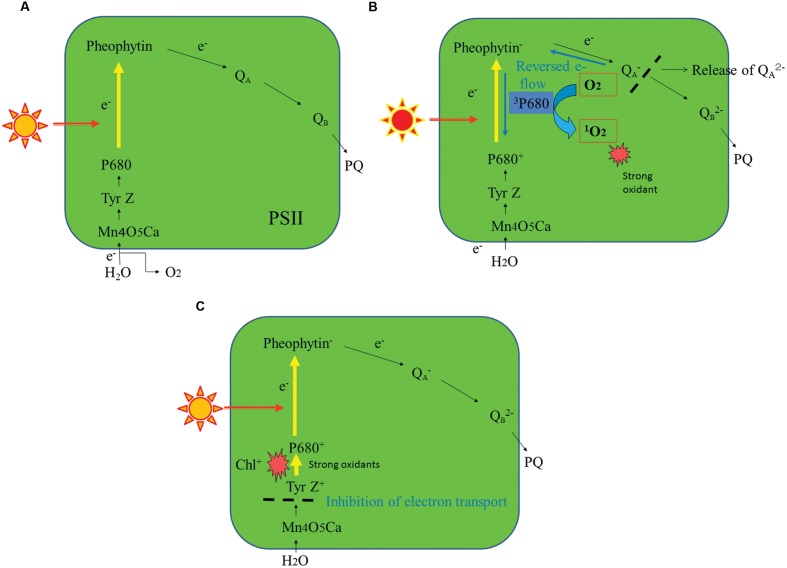
**A schematic representation of the major photoinhibition mechanisms of PSII.** **(A)** A normal electron transport pathway from water to plastoquinone in PSII. **(B)** The acceptor-side photoinhibition mechanism of PSII. **(C)** The donor-side photoinhibition mechanism of PSII. For details, see the text.

The mechanisms of photoinhibition of PSII have been studied extensively at the molecular level (Barber and Andersson, 1992; Aro et al., 1993; Yamamoto et al., 2008). One of them is the acceptor-side photoinhibition, where high light reduces the acceptor side of PSII exceedingly and thereby two electron transport events occur in addition to the usual linear electron transport in PSII (**Figures 2A,B**). The first one is consecutive one-electron donation to oxygen by the reduced electron acceptors of PSII, leading to formation of superoxide anion radicals (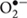). Many components at the acceptor side of PSII can transfer an electron to molecular oxygen: they are the reduced primary electron acceptor pheophytin (Pheo^-^), the reduced plastoquinone acceptor Q_A_^-^, plastosemiquinone and plastoquinol, and cytochrome *b*_559_ (Pospisil, 2009). The second one is a reversed electron flow from Pheo^-^ to the oxidized primary electron donor P680^+^ where the ground-state chlorophylls are excited to the semi-stable triplet state. They react with molecular oxygen and thereby singlet oxygen (^1^O_2_) molecules are formed (Telfer, 2014). Many studies have shown that the ^1^O_2_ thus produced induces the acceptor-side photoinhibition (Barber and Andersson, 1992). The ^1^O_2_ molecules produced here have short lives, indicating that they react immediately at the site of production. Because D1 is close to the ^1^O_2_ production site, the protein is very vulnerable to light stress. The D2 protein (D2) is occasionally photodamaged, but actually D2 is more stable than D1 (Jansen et al., 1999). The site of photodamage in D1 is probably the stroma-exposed DE-loop that connects the membrane spanning helices D and E (Barber and Andersson, 1992; Aro et al., 1993). The acceptor side photoinhibition of PSII has been studied *in vitro* using isolated PSII membranes and PSII complexes (Yamamoto et al., 2008). Contribution of this photoinhibition mechanism under natural light conditions, however, is not clarified yet and requires further study.

Another major mechanism of PSII photoinhibition is the donor-side photoinhibition (**Figure 2C**). Endogenous cationic radicals are produced at the donor-side of PSII by illumination at high or low temperature conditions or under other anomalous conditions such as Cl-depletion or bicarbonate-depletion (Klimov et al., 1997). These cationic radicals are produced because electrons are not transferred efficiently from Mn_4_O_5_Ca complex to the oxidized primary electron donor P680^+^. The P680^+^ and the oxidized form of the secondary electron donor Tyr Z are stabilized and these cationic species may damage D1. For donor-side photoinhibition, weak illumination is enough because the quantum efficiency of photoinhibition is high. The site of the donor-side photoinhibition is suggested to be the lumen-exposed loops of D1 (Barber and Andersson, 1992; Aro et al., 1993). Malfunction of the oxygen-evolving step of PSII under various stress conditions also induces various ROS (Pospisil, 2009). These ROS may react immediately with the neighboring proteins at the donor-side of PSII and inevitably damage them. Like the acceptor-side photoinhibition, the donor-side photoinhibition has been studied extensively *in vitro* (Blubaugh and Cheniae, 1990; Klimov et al., 1990; Yamamoto et al., 2008). However, how much the donor-side photoinhibition contributes to the photoinhibition under natural conditions remains unknown. It was shown previously that illumination of spinach leaves with low light at higher temperature (35–40°C) induced severe photodamage to PSII, and that is one of the rare examples showing donor-side photoinhibition *in vivo* (Ohira et al., 2005).

### Disassembly of the PSII-LHCII Supercomplexes in the Reversible Photoinhibition

**FIGURE 3:**
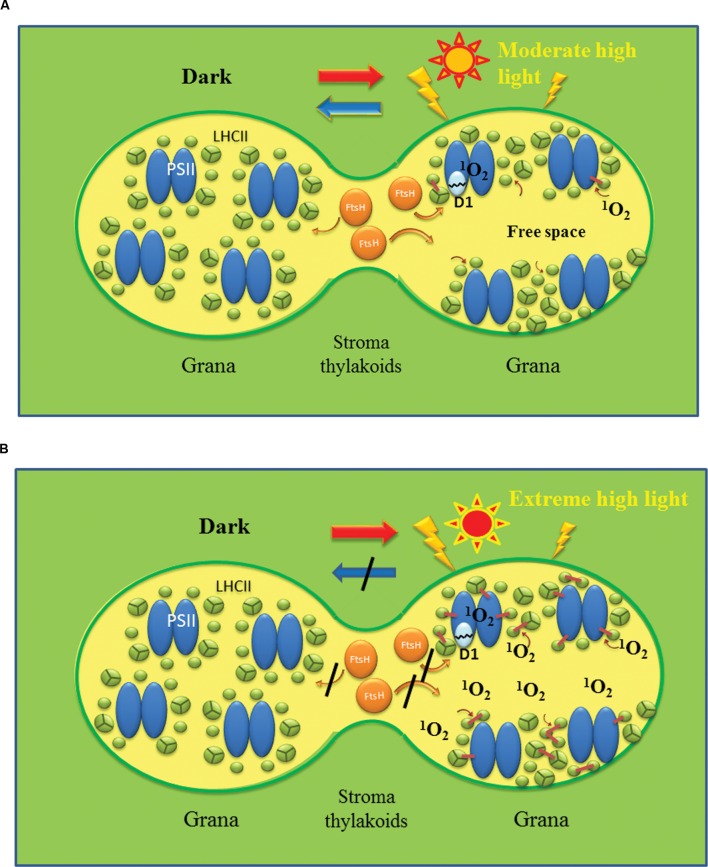
**Shematic models showing reversible and irreversible photoinhibition of PSII.** Reversible photoinhibition **(A)** occurs when thylakoids are irradiated with moderate high light, while irreversible photoinhibition **(B)** occurs when the illumination is extremely strong. In **(A)**, PSII/LHCII complexes are redistributed on the membrane under high light and the photodamaged D1 is removed by FtsH protease and other proteases. Free spaces with high membrane fluidity may be produced at the grana regions to assist the movement of the protein complexes and proteases to remove the damaged D1. PSII activity recovers after the repair of PSII in the dark. By contrast, in **(B)** excessive production of ROS such as ^1^O_2_ through photochemistry of PSII and lipid peroxidation oxidatively damage various proteins and lipids and cause irreversible cross-linking of proteins and lipids. The overall membrane fluidity may decrease and movement of protein components may be hindered significantly. Under these conditions, PSII function and turnover are inhibited completely, and eventually cell death will be brought about.

The disassembly pathway of the photodamaged PSII complex has been clarified considerably by extensive studies for decades (Tikkanen et al., 2008; Yamamoto et al., 2008; Nath et al., 2013; Yamamoto and Yoshioka-Nishimura, 2016). To remove photodamaged D1 from the PSII complex, the PSII-LHCII supercomplex must be disassembled. First the central PSII dimers are dissociated from the surrounding LHCII trimers and minor LHCs, which is followed by disorganization of the PSII dimers containing the photodamaged D1 (Yamamoto et al., 2008). Earlier biochemical analyses showed that a core antenna chlorophyll-binding protein CP43 is released firstly from the PSII complex (Barbato et al., 1992a; Aro et al., 2005). CP43 is one of the several phosphoproteins in the core of PSII. Other phosphoproteins in the PSII core are D1, D2, the PsbH subunit and the TSP9 protein (Bennett, 1980; Vener, 2007). The PSII core proteins are strongly phosphorylated under high light conditions (Rintamaki et al., 1996), and the phosphorylation is mainly carried out by STN8 kinase (Bonardi et al., 2005) although STN7 kinase also shows minor activity (Vainonen et al., 2005). The use of stn7 and 8 mutants has revealed a clear relationship between protein phosphorylation and disorganization of the PSII complex (Tikkanen and Aro, 2012). Phosphorylation of the core part of the PSII complex under excessive illumination was shown to help disassembly of the damaged PSII complex (Tikkanen et al., 2008; Fristedt and Vener, 2011). After CP43 is removed from the PSII complex, the damaged and phosphorylated D1 is dislocated and moves to the membrane area, where D1 is dephosphorylated by a phosphatase(s) and then digested by a protease(s). Phosphatases involved in these steps have been identified (Vener et al., 1999; Sirpio et al., 2008; Samol et al., 2012). To support the dynamic changes in the distribution and orientation of PSII-LHCII supercomplexes and of the components related to the repair of the photodamaged PSII on the thylakoids described above, membrane fluidity is essential. The fluidity ensures lateral diffusion of the related membrane components (Yamamoto et al., 2013, 2014; Kirchhoff, 2014; **Figure 3A**).

### Degradation of the Damaged D1 in the Reversible Photoinhibition

Because accumulation of photodamaged proteins in PSII complexes is a potential hazard to PSII, the damaged D1 must be removed promptly by specific proteases. Currently two proteases are the candidates that target the damaged D1: ATP-dependent zinc-metalloprotease FtsH (filamentation temperature sensitive H; Silva et al., 2003; Komenda et al., 2006) and ATP-independent serine protease Deg (degradation of periplasmic proteins; Itzhaki et al., 1998; Kapri-Pardes et al., 2007; Sun et al., 2007; Kato et al., 2012). FtsH is a thylakoid membrane-bound processive endopeptidase, while Deg is a thylakoid lumen-localized endopeptidase. Of four Deg proteases present in *Arabidopsis* chloroplast, Deg1, 5 and 8 are located in the lumen, whereas Deg2 is attached to the stromal side of the thylakoids. The damaged D1 is recognized by either of the two proteases, and the recognition is likely to be defined by the location of the damaged site, namely the donor side (luminal side) or the acceptor side (stromal side) of D1. These proteases cleave the damaged D1 into primary fragments (Yoshioka-Nishimura and Yamamoto, 2014). The primary fragments are further digested to smaller fragments and they are subsequently broken down to amino acids. The details of the proteolysis are not clear yet.

**FIGURE 4:**
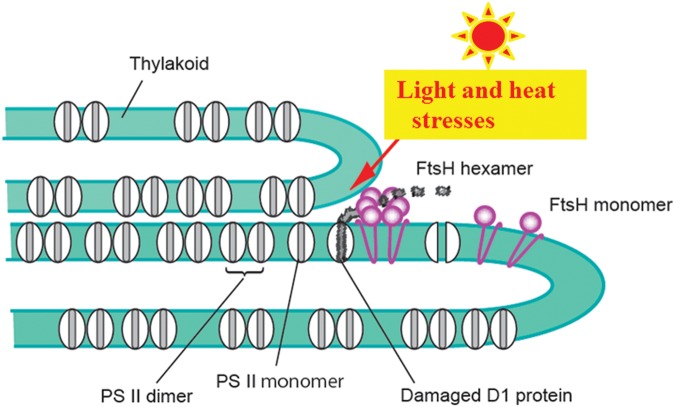
**Photo-and heat-induced damage to PSII and removal of the damaged D1 by FtsH proteases.** To simplify the model, only the PSII complexes and FtsH proteases are shown. For details, see the text.

Where in the thylakoid membranes the degradation of D1 occurs remains to be clarified. The thylakoid membranes are abundant with various protein complexes including PSI, PSII, cytochrome *b*_6_/*f* and ATP synthase, and are densely packed with these components (Armond et al., 1977; Albertsson, 2001; Allen and Forsberg, 2001). Under these conditions how D1 degradation occurs is our primary concern. The degradation has been thought to take place at the stroma thylakoids where membrane-bound oligomeric proteases can easily reach the damaged proteins in the grana where PSII complexes are abundant. To make this access possible, PSII complexes retaining the damaged D1 must move from the grana core to the stroma thylakoids even though the grana are crowded with PSII complexes and other proteins. In a recent study, however, it was shown that the grana margins and also the grana end membranes are the actual sites of D1 degradation (Yoshioka et al., 2010; **Figure 4**). The PSII complexes containing damaged D1 do not need to move through the grana to the degradation sites. When D1 degradation occurs at the luminal side by lumen-localized Deg proteases, the grana stack will not block access of the lumen protease to the damaged D1, although extrinsic proteins such as PsbO, P and Q may shield D1 from the proteases. In such a case, release of these extrinsic proteins from PSII is necessary for dislocation and subsequent degradation of D1 (Henmi et al., 2004).

Regarding the above results, there is an argument that the grana margins and the grana end membranes are crowded with PSI and other complexes, and that these conditions may hinder the D1 turnover at the suggested areas. The protein density in the stroma thylakoids is assumed to be lower than that in the grana (Kirchhoff, 2014), and if this is also the case with the grana margins and the grana end membranes, the above proposal may be valid. At present, the data showing exact protein packing densities at the grana margins and the grana end membranes as well as other areas are not available (Kirchhoff, 2014), and we have to await the reevaluation of these results in the future.

### *De novo* Synthesis of D1, Insertion of D1 to the D1-Depleted PSII Complex, and Reassembly of the PSII Complex

After degradation of D1, a new copy of D1 is synthesized on thylakoid-bound ribosomes (Baena-Gonzalez and Aro, 2002). The signal that induces expression of the *psbA* gene encoding D1 is not clear, but ^1^O_2_ and the other ROS produced by high light are good candidates (Laloi et al., 2007; Krieger-Liszkay et al., 2008; Fischer et al., 2013). On translation of D1, the elongating polypeptide chain is directly inserted into the thylakoid membrane and associates with the PSII core. The elongation step of D1 translation has been suggested to be susceptible to ROS and that the inhibition of D1 repair is the primary cause of photoinhibition (Nishiyama et al., 2004).

Formation of a multisubunit PSII complex has been shown to start with accumulation of D2, followed by incorporation of D1. The antenna chlorophyll-binding protein CP47 is then attached to form a PSII core complex (Baena-Gonzalez and Aro, 2002; Komenda et al., 2004). The α-subunit of cytochrome *b*_559_ also appears at an early stage of PSII assembly (Hashimoto et al., 1993; Morais et al., 1998). Considering these facts, intactness of D2 and the other core subunits during photoinhibition is important for subsequent PSII assembly. As already described, CP43 is removed first when the PSII complex disassembles under light stress, and the converse may be true when the PSII complex is organized: CP43 is the last protein to complete formation of the PSII core in the reassembly process. Extrinsic proteins of PSII are partly located in the pool in the thylakoid lumen as free forms (Hashimoto et al., 1996) and they bind to the PSII core at specific timings in the assembly process (Hashimoto et al., 1993, 1997). This elaborate process of D1 turnover ensures quick restoration of PSII activity after its impairment by photoinhibition. Thus the level of the reversible photoinhibition is determined by the balance of the damage and repair of D1.

## Irreversible Photoinhibition

When chloroplasts are irradiated with extreme high light, irreversible aggregation between the damaged D1 and neighboring proteins occurs in PSII (Barbato et al., 1992b; Ishikawa et al., 1999; Yamamoto et al., 2008). Strong illumination accelerates protein crosslinking (aggregation) and the protein aggregates may prevent smooth movement of proteins and lipids on the membrane. Since membrane fluidity and protein movement are essential for homeostasis of PSII, accumulation of these protein aggregates may induce photoinhibition of PSII that cannot be repaired (Yamamoto et al., 2013, 2014). Hereafter, this kind of photoinhibition is referred to as ‘*irreversible photoinhibition.*’ The protein aggregation corresponds to covalent cross-linking between the photodamaged D1 and the neighboring polypeptides, including D2, CP43, and the α-subunit of cytochrome *b*_559_ (Ishikawa et al., 1999; Yamamoto, 2001; Henmi et al., 2003; Yamamoto et al., 2008). In some cases, the crosslinking sites of the PSII proteins were determined (Barbato et al., 1995; Mizusawa et al., 2003). Although the irreversible protein cross-linking was mostly determined with the proteins such as D1, D2, CP43, CP47, cytochrome *b*_559_, and LHCII subunit proteins because antibodies against these proteins are available, it is likely that the protein cross-linking is a general phenomenon observable in many proteins existing in the thylakoid membranes, including those participating in the damage-repair cycle of PSII. As the previous chemical model experiments show clearly, oxidative damage to proteins is manifested as protein cross-linking and protein cleavage, as well as protein carbonylation (Berlett and Stadtman, 1997; Halliwell and Gutteridge, 2007). The irreversible photoinhibition may eventually cause dysfunction of chloroplasts and cell death (**Figure 3B**).

## Heat Inactivation of Photosystem II

Inactivation of PSII is also induced by moderate heat stress on the thylakoids such as 40°C for 30 min. The results of the heat-inactivation of PSII are quite similar to those of the reversible and irreversible photoinhibition of PSII in many aspects. Importantly, D1 is susceptible to heat-inactivation, and degradation and aggregation of the protein occur (Yoshioka et al., 2006; Komayama et al., 2007). The cause of inactivation of PSII seems to be not denaturation of PSII components by heat but damage by ROS produced by heat stress. Indeed, production of ^1^O_2_ and hydroxyl radical (HO^•^) by moderate heat stress on spinach thylakoids was demonstrated by EPR spin trapping, and oxidation and cleavage of D1 was observed in parallel (Yamashita et al., 2008). The cleavage is probably catalyzed by a protease(s) and not by ROS. FtsH is a good candidate for the protease (Yoshioka et al., 2006; Yamashita et al., 2008; **Figure 4**). However, it is not clear whether the primary cleavage products are further broken down under moderate heat stress. The aggregates of D1, which were detected in parallel with D1 cleavage, showed molecular sizes similar to those observed under photoinhibition (Komayama et al., 2007). It was demonstrated that lipid peroxide-related substances as well as ROS are produced in the thylakoids by the moderate heat stress, and they have been thought to be responsible for the protein aggregation as described below (Chan et al., 2012).

## Membrane Lipids and Membrane Fluidity

Lipids are the necessary basis of structure of the thylakoid membranes and of various reactions in photosynthesis (Wada and Murata, 2010). Lipids form a matrix for proper functioning of large pigment-protein complexes such as PSI and PSII as well as many other low molecular weight components in the thylakoids. Free-moving lipids, or bulk lipids, have certain fluidity determined by degree of unsaturation of constituent fatty acids and ambient temperature. The membrane fluidity regulates chemical and biological reactions in the thylakoids. In addition to these free-moving lipids, there are the so-called “bound lipids” which are closely associated with membrane proteins: they are located either at the membrane-exposed surface of protein complexes or at well-defined positions of the proteins (Loll et al., 2007; Mizusawa and Wada, 2012).

In the thylakoids of higher plant chloroplasts, glyceroglyco lipid monogalactosyldiacylglycerol (MGDG) and digalactosyl diacylglycerol (DGDG) are the major lipids, making up 50–60 and 20–30 mol percent of total lipids, respectively (Somerville et al., 2000). As minor lipids, glycerophospholipid phosphatidylglycerol (PG) and another glyceroglycolipid sulfoquinovosyldiacylglycerol (SQDG) are present (Dorne et al., 1990). Importantly the fatty acids of these membrane lipids are highly unsaturated. Poly unsaturated fatty acids 18:3 (18 is the number of carbon atom and 3 is the number of double bond in the acyl chain) and 16:3 account for ∼70 mol percent of the fatty acids in the thylakoids and >90 mol percent of the fatty acids in MGDG (Somerville et al., 2000). Other lipids DGDG, PG, and SQDG are comprised of 18:3, and 16:0 fatty acids. The presence of polyunsaturated fatty acids (PUFA) is the basis of the membrane fluidity. The lipid and protein diffusion coefficients in the thylakoid membranes obtained in the past studies are nicely summarized in a recent review (Kirchhoff, 2014). Both the earlier and recent studies show rather small diffusion coefficients in the overall thylakoids (Rubin et al., 1981; Kirchhoff et al., 2008), reflecting the highly crowded nature of the membranes, but information on the local membrane fluidity is not available yet.

## Lipid Peroxidation Under Light Stress and Heat Stress

Although PUFA are important for membrane fluidity, they also become a target of lipid peroxidation (Halliwell and Gutteridge, 2007). The lipid peroxidation occurs when the thylakoids are exposed to various oxidative stresses including light stress and heat stress (Apel and Hirt, 2004; Triantaphylides et al., 2008; Yamashita et al., 2008; Triantaphylides and Havaux, 2009; Chan et al., 2012; Vass, 2012). Lipid peroxidation is a process in which lipids are oxidatively converted to lipid peroxides (LOOHs; Frankel, 1980). The process consists of initiation, propagation, and termination steps. Through these steps lipid radicals and secondary products including aldehydes and ROS are generated (Esterbauer et al., 1991). It was shown with spinach thylakoids that lipid peroxidation induced by high light or moderate heat stress causes damage to D1 and LHCII as well (Yamashita et al., 2008; Chan et al., 2012), and the damage was suggested to be due to ROS produced during lipid peroxidation and the secondary products of lipid peroxidation.

It is important to note that lipid peroxidation possibly affect membrane fluidity (Yamamoto et al., 2013). Many reactive lipid peroxidation products cause protein oxidation, protein cleavage and protein–protein crosslinking. Lipid-protein and lipid–lipid crosslinkings are also probable. Indeed, irreversible protein aggregation was observed with spinach thylakoids and PSII membranes when lipid peroxidation was induced by high light or moderate heat treatment (Chan et al., 2012). These crosslinking or aggregation steps of proteins and lipids should significantly affect the total and local membrane fluidity in the thylakoids (Yamamoto et al., 2013; **Figure 3B**).

## Possible Change in the Normal Dynamics of the Thylakoids Under Light Stress and Heat Stress

Thylakoid dynamics, which is dependent on membrane fluidity, is important for supporting the functions of chlorophyll-protein complexes including PSII/LHCII supercomplexes in the thylakoids. The normal thylakoid structure and dynamics may be disturbed by light and heat stresses. Heat stress-induced dissociation of supercomplexes in the thylakoid membranes and redistribution and randomization of the complexes were reported earlier based on electron microscopic evidences (Armond et al., 1980). After the stress, the thylakoid membranes attain a new equilibrium to adapt to the stress conditions. Lateral movement of proteins and lipids enable the rearrangement of the chlorophyll-protein complexes on the thylakoids, and therefore the molecular movement is the essential basis of the avoidance and tolerance mechanisms of PSII against light and heat stresses (Yamamoto et al., 2014). The thylakoids, in particular the grana, are crowded with large protein complexes and smaller proteins, and well-coordinated movement of these components under various light and temperature conditions on the membranes is crucial for keeping the activity of the thylakoids. Phosphorylation of PSII was shown to regulate the stacking and unstacking of the thylakoids in *Arabidopsis* (Fristedt et al., 2009). Phosphorylation and de-phosphorylation of the PSII proteins are expected to play a crucial role in the regulation of distribution of proteins on the thylakoids, and kinases and phosphatases responsible for these reactions are of course workable when they move easily on the thylakoids.

**FIGURE 5:**
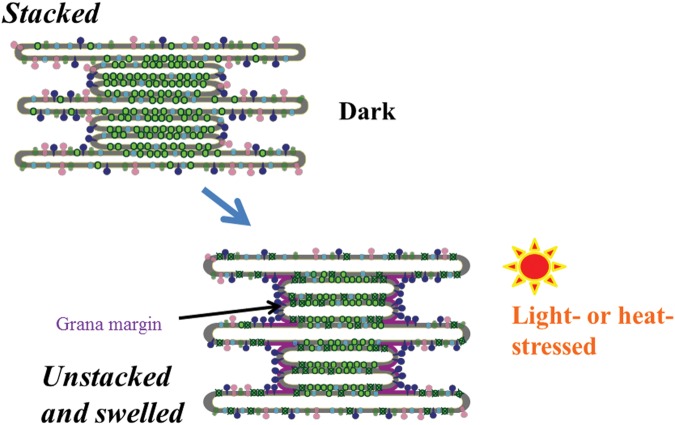
**A schematic diagram of light- or heat-induced unstacking of the thylakoids and swelling of thylakoid lumen.** Light green particles in the grana represent PSII/LHCII complexes. Dark blue particles represent FtsH proteases, which recognize the damaged PSII complexes at the grana margins, stroma thylakoids and grana end membranes, and carry out degradation of the damaged D1. Purple, light green and light blue particles represent ATP synthase, PSI complex, and cytochrome *b*_6_/*f* complex, respectively. Because the exact protein packing densities in each membrane compartment such as stroma thylakoids, grana, and grana margins are not known yet, the diagram shows only a simplified image in regard to the distribution of protein complexes.

In addition to this fine-tuning of the distribution of proteins on the thylakoids, changes in the structure of the thylakoids at a larger scale, such as stacking/unstacking and shrinkage/swelling of the thylakoids, were also shown (Khatoon et al., 2009; Herbstova et al., 2012; Yamamoto et al., 2013; Puthiyaveetil et al., 2014; Yoshioka-Nishimura et al., 2014; **Figure 5**). The necessity of thylakoid unstacking under light stress was first postulated when the details of D1 degradation is examined. In the degradation of the damaged D1 by FtsH protease, it is likely that extrusion of the hydrophilic part of FtsH limits its movement from the stroma thylakoids to the grana. FtsH protease, which is anchored to the thylakoid membrane, has a large hydrophilic domain at the C-terminus exposed to the stroma. It is 6.5 nm in height, and since the width of the stromal gap between the two adjacent grana membranes is only ∼3.5 nm (Daum et al., 2010; Kirchhoff et al., 2011), the protease must be excluded from the grana thylakoids. It was shown from digitonin treatment of isolated spinach thylakoids and subsequent centrifugation (Chow et al., 1980) that grana stacking decreases irreversibly under strong light (Khatoon et al., 2009). In accordance with that, transmission electron microscopy (TEM) showed that after intense illumination of spinach leaves thylakoid membranes bent outward at both ends of the grana and the gap between the grana margins widened (Yamamoto et al., 2013; Yoshioka-Nishimura et al., 2014). A similar high light-induced bending of the thylakoids was also observed with *Arabidopsis* leaves (Puthiyaveetil et al., 2014). Immunoelectron microscopy showed that the FtsH proteases labeled with gold particles were observed in the stroma thylakoids and the grana margins in the dark, whereas subsequent strong illumination increased immunogold in the grana stacks and degradation of D1 occurred in parallel (Yoshioka-Nishimura et al., 2014). It was suggested from these results that under light stress the FtsH proteases migrate to the grana core and degrade damaged D1 in the grana. In addition, 3D tomography has revealed that when spinach thylakoid membranes are exposed to strong light for an hour, the stacked grana loosen not only at the grana margins but also throughout the entire thylakoid membranes (Yoshioka-Nishimura et al., 2014). The unstacking of the thylakoids may release the constraints on the movement of PSII complexes and other proteins and facilitate their migration on the thylakoids. It is likely that the unstacking of the thylakoid membranes starts from the grana margins, and it expands into the whole areas of thylakoid stacks eventually. It is also pointed out that partial unstacking of the thylakoids occurs not only at the marginal regions but also in the middle of the grana stack due to local fluctuation, which may facilitate the mobility of the complexes in the grana (Garab, 2014).

For the study of membrane fluidity, many tools have been employed. In early studies, fluorescence polarization of a fluorescence probe diphenylhexatrien (DPH) was used to monitor membrane fluidity of spinach thylakoids (Yamamoto et al., 1981). Even though the method was shown to have certain limitations (Ford and Barber, 1983), DPH and its derivatives are often used to study relationship between membrane fluidity and light/heat stresses (Velitchkova et al., 2001, 2009; Yamamoto et al., 2013). Electron paramagnetic resonance was also used for this purpose (Ford and Barber, 1983; Tardy and Havaux, 1997; Kota et al., 2002). Fluorescence recovery after photobleaching (FRAP) is a more sophisticated method using confocal fluorescence microscopy (Sarcina et al., 2006; Goral et al., 2010; Johnson et al., 2011; Herbstova et al., 2012). Using FRAP, many useful data have been obtained showing the dynamic aspects of thylakoids during light stress. However, more easy, reliable and quantitative methods are necessary to estimate both the total and local membrane fluidity. Visualization of the movement of membrane components on the thylakoid membranes during the light stress and heat stress may be the final goal of the studies now in this research field.

## Conclusion and Future Perspectives

**FIGURE 6:**
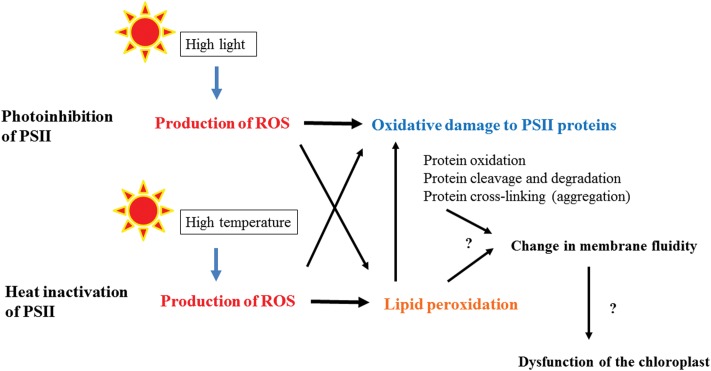
**A flow chart showing the photoinhibition of PSII under high light and heat-inactivation of PSII under high temperature.** Under the light stress and heat stress, ROS are produced at PSII or in the thylakoid membranes, which cause oxidative damage to PSII directly or through lipid peroxidation. Severe damages may be manifested as protein–protein, protein-lipid, and lipid–lipid cross-linking and they affect the total and local membrane fluidity of the thylakoids. Hindrance of efficient movement of the proteins in the thylakoids may results in dysfunction of the chloroplasts and finally cell death.

Light stress and heat stress induce inhibition of PSII activity through oxidative damage to PSII proteins. The reaction-center binding D1 protein of PSII is most prone to the oxidative damage. In the photoinhibition of PSII under high light, ROS produced in PSII may injure D1, while in heat-inactivation of PSII, D1 seems to be damaged by lipid peroxidation occurring near PSII. Before inactivation of PSII starts and during the inactivation as well, many molecular mechanisms work to avoid and tolerate the stresses (Li et al., 2009; Jahns and Holzwarth, 2012). Anti-oxidants and detoxifying enzymes are effective tools in the chloroplasts to cope with the stresses (Asada, 2006), whereas the protective processes in the thylakoids are largely dependent on the membrane fluidity of the thylakoids. The fluidity may change upon light and heat stresses by disturbing the ordered distribution of proteins and protein complexes. Stress-induced thylakoid unstacking may become a force to increase the membrane fluidity. However, when the stresses are exceedingly strong, the membrane fluidity may be decreased by light- or heat-induced crosslinking of proteins and lipids in the thylakoids (**Figure 6**). Thus, thylakoid membrane fluidity seems to work as an important mediator of quality control of PSII under light stress and heat stress.

The chemical basis of protein oxidation and subsequent protein cleavage and crosslinking (aggregation) has been studied extensively using artificially generated ROS such as hydroxyl radicals produced by radiolysis of water and model proteins such as bovine serum albumin (Berlett and Stadtman, 1997; Stadtman and Levine, 2000; Halliwell and Gutteridge, 2007). The mechanism of lipid peroxidation has also been investigated in various model systems (Frankel, 1980; Halliwell and Gutteridge, 2007). The results obtained from these chemical studies give us valuable hints to think about the effects of light and heat stresses on PSII. However, it is apparent that there is still a significant gap between the chemical data obtained in the model experiments and the data obtained with the complex biological systems. To further the study on the molecular dynamics of the thylakoids, more elaborated and quantitative methods should be introduced to estimate protein oxidation, lipid peroxidation, and related subsequent processes in the thylakoid membranes under light- and heat stressed conditions. When these conditions are attained, we have to try to determine the chemical basis of the observed biological phenomena more exactly. In this regard, development of quantitative assay methods to measure total and local membrane fluidity may be crucial for the future advance of this research subject. Visualization of the molecular events in the thylakoid membranes under light stress and heat stress is also important.

## Author Contributions

The author confirms being the sole contributor of this work and approved it for publication.

## Conflict of Interest Statement

The author declares that the research was conducted in the absence of any commercial or financial relationships that could be construed as a potential conflict of interest.
